# Cisplatin-incorporating polymeric micelles (NC-6004) can reduce nephrotoxicity and neurotoxicity of cisplatin in rats

**DOI:** 10.1038/sj.bjc.6602772

**Published:** 2005-09-13

**Authors:** H Uchino, Y Matsumura, T Negishi, F Koizumi, T Hayashi, T Honda, N Nishiyama, K Kataoka, S Naito, T Kakizoe

**Affiliations:** 1 Investigative Treatment Division, National Cancer Center Research Institute East, 6-5-1 Kashiwanoha, Kashiwa, Chiba 277-8577, Japan; 2 NanoCarrier Co., Ltd, 5-4-19 Kashiwanoha, Kashiwa, Chiba 277-0882, Japan; 3 Department of Anatomy and Histology, Fukushima Medical University School of Medicine, 1-Hikariga-oka, Fukushima, Fukushima 960-1247, Japan; 4 Department of Materials Science and Engineering, Graduate School of Engineering, The University of Tokyo, 7-3-1 Hongo, Bunkyo-ku, Tokyo 113-8656, Japan; 5 Department of Urology, Graduate School of Medical Sciences, Kyushu University, 3-1-1 Maidashi, Higashi-ku, Fukuoka, Fukuoka 812-8582, Japan; 6 National Cancer Center, 5-1-1 Tsukiji, Chuo-ku, Tokyo 104-0045, Japan

**Keywords:** cisplatin, polymeric micelle, EPR effect, neurotoxicity

## Abstract

In spite of the clinical usefulness of cisplatin (CDDP), there are many occasions in which it is difficult to continue the administration of CDDP due to its nephrotoxicity and neurotoxicity. We examined the incorporation of CDDP into polymeric micelles to see if this allowed the resolution of these disadvantages. Cisplatin was incorporated into polymeric micelles through the polymer–metal complex formation between polyethylene glycol poly(glutamic acid) block copolymers and CDDP (NC-6004). The pharmacokinetics, pharmacodynamics, and toxicity studies of CDDP and NC-6004 were conducted in rats or mice. The particle size of NC-6004 was approximately 30 nm, with a narrow size distribution. In rats, the area under the curve and total body clearance values for NC-6004 were 65-fold and one-nineteenth the values for CDDP (*P*<0.001 and 0.01, respectively). In MKN-45-implanted mice, NC-6004 tended to show antitumour activity, which was comparable to or greater than that of CDDP. Histopathological and biochemical studies revealed that NC-6004 significantly inhibited the nephrotoxicity of CDDP. On the other hand, blood biochemistry revealed transient hepatotoxicity on day 7 after the administration of NC-6004. Furthermore, rats given CDDP showed a significant delay (*P*<0.05) in sensory nerve conduction velocity in their hind paws as compared with rats given NC-6004. Electron microscopy in rats given CDDP indicated the degeneration of the sciatic nerve, but these findings were not seen in rats given NC-6004. These results were presumably attributable to the significantly reduced accumulation of platinum in nerve tissue when NC-6004 was administered (*P*<0.05). NC-6004 preserved the antitumour activity of CDDP and reduced its nephrotoxicity and neurotoxicity, which would therefore seem to suggest that NC-6004 could allow the long-term administration of CDDP where caution against hepatic dysfunction must be exercised.

Cisplatin (*cis*-dichlorodiammineplatinum (II): CDDP) is a key drug in the chemotherapy for cancers, including lung, gastrointestinal, and genitourinary cancer (Roth, 1996; Boulikas and Vougiouka, 2004). However, we often find that it is necessary to discontinue treatment with CDDP due to its adverse reactions, for example, nephrotoxicity and neurotoxicity, despite its persisting effects (Pinzani *et al*, 1994). Platinum (Pt) analogues, for example, carboplatin and oxaliplatin (Cleare *et al*, 1978), have been developed to date to overcome these CDDP-related disadvantages. Consequently, these analogues are becoming the standard drugs for ovarian cancer (du Bois *et al*, 2003) and colon cancer (Cassidy *et al*, 2004). However, those regimens including CDDP are considered to constitute the standard treatment for lung cancer, stomach cancer, testicular cancer (Horwich *et al*, 1997), and urothelial cancer (Bellmunt *et al*, 1997). Therefore, the development of a drug delivery system (DDS) technology is anticipated, which would offer the better selective accumulation of CDDP into solid tumours while lessening its distribution into normal tissue.

Drug delivery system targeting involves two concepts: active targeting and passive targeting. Active targeting aims drug targeting through antigen–antibody reactions and specific bindings between molecules, for example, receptor and ligand. On the other hand, passive targeting is an approach in which the drug accumulates in tumour tissue using the pathophysiological characteristics of solid tumours such as the hyperplasia of tumour vasculature which generally occurs in solid tumours, but which is not seen in a comparable way in lymph nodes. Marked vascular hyperpermeability is also found in the tumour vasculature, and the combination of hyperplasia and hyperpermeability facilitate the extravasation of high-molecular-weight polymers or nanoparticles, which are less prone to leak from intact vasculature, and which can be retained in solid tumour tissue for a longer time (enhanced permeability and retention effect (EPR) effect) (Matsumura and Maeda, 1986; Maeda and Matsumura, 1989; Maeda, 2000, 2001). This effect allows passive targeting of macromolecules with a high blood retention profile into the site of tumour.

Simple polymerisation only is not sufficient to bring about the EPR effect, and strategies are also required to suppress trapping by the reticuloendothelial system (RES) and to enhance the blood retention profile (Klibanov *et al*, 1990, 1991; Allen, 1994; Gabizon *etal*, 1996; Lasic, 1996). Polyethylene glycol-tagged liposomal adriamycin (Doxil®) has recently been reported as a clinical success (Orditura *et al*, 2004). We have recently been conducting research dedicated to the development of polymeric micelles capable of incorporating anticancer drugs (Yokoyama *et al*, 1990, 1991, 1999). The Phase I clinical trial of adriamycin-incorporating polymeric micelles has been completed (Matsumura *et al*, 2004). Furthermore, in an animal model, the plasma and tumour area under the curve (AUC) values for taxol-incorporating polymeric micelle (NK105) showed 85- and 25-fold increases, respectively, as compared with those for taxol. Therefore, NK105 showed significant enhancement (*P*<0.001) of the antitumour activity of free taxol and a significant reduction (*P*<0.05) in its neurotoxicity (Hamaguchi *et al*, 2005). Based on these results, the Phase I clinical trial of NK105 is currently being conducted at the National Cancer Center Hospital, Tokyo. We have also been conducting research dedicated to the development of CDDP-incorporating polymeric micelles and have made a number of improvements, in the *in vivo* antitumour activity, reduction of nephrotoxicity, particle size, and particle size distribution as variables (Nishiyama and Kataoka, 2001; Nishiyama *et al*, 2001). Consequently, we discovered that block copolymers, which react with CDDP, acquire a long blood retention profile with the use of polyethylene glycol poly(glutamic acid) block copolymers (PEG–P(Glu)) (Nishiyama *et al*, 2003). In the present study, we used the final development of the technology to prepare CDDP-incorporating polymeric micelles (NC-6004) in an attempt to investigate the following objectives: (1) calculation of pharmacokinetic (PK) parameters in a detailed PK study of CDDP and NC-6004 in rats; (2) a comparison between CDDP and NC-6004 with respect to their antitumour activity in a human cancer cell line; and (3) a detailed comparison between CDDP and NC-6004 with respect to nephrotoxicity and neurotoxicity, which constitute the dose-limiting factors of CDDP.

## MATERIALS AND METHODS

### Materials

Cisplatin was purchased from WC Heraeus GmbH & Co., KG (Hanau, Germany). *γ*-Benzyl-L-glutamate *N*-carboxy anhydride was purchased from a supplier. *N,N*-dimethylformamide and 3-(4,5-dimethylthiazol-2-yl)-2,5-diphenyltetrazolium bromide were purchased from Wako Pure Chemical Co., Inc. (Osaka, Japan). *α*-Methoxy-*ω*-aminopropyl polyethylene glycol (CH_3_O–PEG–CH_2_CH_2_CH_2_–NH_2_; MW=12 000) was purchased from NOF Corporation (Tokyo, Japan).

Following cell lines, MKN-45, MKN-28, EJ-1, J82, MBT-2, colo201, colo320, HT-29, A549, EBC-1, PC-14, and MCF-7 cells were purchased from the American Type Culture Collection.

Female BALB/*c nu/nu* mice were purchased from SLC (Shizuoka, Japan). Female Sprague–Dawley rats were purchased from Charles River Japan (Kanagawa, Japan). All animal procedures were performed in compliance with the guidelines for the care and use of experimental animals, which had been drawn up by the Committee for Animal Experimentation at the National Cancer Center; these guidelines meet the ethical standards required by law and also comply with the guidelines for the use of experimental animals in Japan and the UKCCCR guidelines (UKCCCR, 1998).

### Preparation of PEG-P(Glu) and preparation of CDDP-incorporating polymeric micelles (NC-6004)

Polyethylene glycol–P(Glu) block copolymers were synthesised according to the slightly modified procedure of the previously reported synthetic method of PEG-P(Asp) (Nishiyama and Kataoka, 2001). *γ*-Benzyl L-glutamate *N*-carboxy anhydride was polymerised in *N,N*-dimethylformamide, initiated with the NH_2_ amino group of CH_3_O–PEG–CH_2_CH_2_CH_2_NH_2_, to obtain PEG–poly(*γ*-benzyl L-glutamate) block copolymers (PEG–PBLG). The polymerisation degree of PBLG was determined to be 40 by comparing proton ratios between PEG (−OCH_2_CH_2_–: *δ*=3.7 p.p.m.) and phenyl groups of PBLG (−CH_2_C_6_H_5_: *δ*=7.3 p.p.m.) in ^1^H NMR measurement (Mercury plus 300 (Varian Technologies); solvent: DMSO-d_6_; and temperature: 25°C). The benzyl group was deprotected by mixing with 0.5 N NaOH at ambient temperature to obtain PEG–P(Glu) as a sodium salt.

Cisplatin-incorporating polymeric micelles (NC-6004) were prepared according to the slightly modified procedure of the previously reported synthetic method of CDDP-incorporating polymeric micelles (Nishiyama *et al*, 2003). Briefly, the sodium salt of PEG–P(Glu) and CDDP were dissolved in distilled water ([Glu]=4.7 mmol l^−1^; [CDDP]/[Glu]=1.0) and were allowed to react for 72 h. NC-6004 thus prepared was purified with ultrafiltration (molecular weight cutoff size: 100 000). The size distribution of NC-6004 was evaluated by dynamic light scattering (DLS) at 23°C using the NICOMP 380 ZLS particle sizer (Particle Sizing Systems, Santa Barbara, CA).

### Release of CDDP from NC-6004 dissolved in saline

NC-6004 was dissolved in saline and was then incubated at 37°C. In all, 80 *μ*l of the solution was then harvested at 3, 6, 24, and 96 h after the onset of incubation. The release of CDDP from NC-6004 in the solution harvested at 37°C was quantified by gel permeation chromatography (column: Waters Ultrahydrogel 500 (*ϕ*7.8 × 300 mm); Waters GPC system equipped with a UV detector (310 nm); and eluent: 10 mmol l^−1^ phosphate-buffered 50 mmol l^−1^ NaCl solution).

### *In vitro* cytoxicity

Various human cancer cell lines were evaluated in the present study. The cell lines were maintained in monolayer cultures in Dulbecco’s modified Eagle’s medium containing 10% (v v^−1^) focal calf serum and 600 mg l^−1^ glutamine. WST-8 Cell Counting kit-8 (Dojindo, Kumamoto, Japan) was used for cell proliferation assay. In all, 2000 cells of each cell line in 90 *μ*l of culture medium were plated in 96-well plates and were then incubated for 24 h at 37°C. Serial dilutions of CDDP and NC-6004 in a volume of 10 *μ*l were added, and the cells incubated for 48 or 72 h. All dates were expressed as mean±s.e. of triplicate of the date triplicate cultures. The data were then plotted as a percentage of the data from the control cultures, which were treated identically to the experimental cultures, except that no drug was added.

### Pharmacokinetics and pharmacodynamics of CDDP and NC-6004

Under isoflurane anaesthesia, a polyethylene catheter was inserted into the right internal jugular vein of female Sprague–Dawley female rats. Rats (*n*=3) were given a single intravenous (i.v.) injection of CDDP (5 mg kg^−1^) or NC-6004 (an equivalent dose of 5 mg kg^−1^ CDDP) via the tail vein. At 5, 15, and 30 min, as well as at 1, 4, 12, 24, and 48 h after injection of each drug, blood (0.2 ml) was collected into a heparinised microtube via the polyethylene catheter. The blood samples were centrifuged (1000 **g**) for 10 min at room temperature to obtain the plasma. The plasma samples were stored below −80°C until the analysis. In a tissue distribution study, rats were injected i.v. with CDDP (5 mg kg^−1^) or NC-6004 (an equivalent dose of 5 mg kg^−1^ CDDP) via the tail vein, and were then killed in groups of three animals at 10 min, at 1, 6, 24, and 48 h, and on day 7 day after injection of each drug under intraperitoneal pentobarbital anaesthesia (50 mg kg^−1^). Various organs (kidney, liver, spleen, heart, lung, small intestine, colon, and stomach) were dissected. The organ samples were stored below −80°C until the analysis. Female BALB/*c* mice were inoculated subcutaneously on the back with 10^6^ MKN-45 cells (UKCCCR, 1998). After 10 days, when the tumour size had reached approximately 50 mm^2^, mice were injected i.v. with CDDP (5 mg kg^−1^) or NC-6004 (an equivalent dose of 5 mg kg^−1^ CDDP) via the tail vein and were then killed in groups of three animals at 10 min, at 1, 6, 24, and 48 h, and on day 7 after injection of each drug. The tumours were dissected and stored below −80°C until the analysis. The plasma samples were diluted with 0.1 N HCl, vortexed, and analysed for elemental Pt by frameless atomic absorption spectrophotometry (FAAS). The tissue samples were decomposed by heating in concentrated nitric acid, evaporated to dryness, and redissolved in 0.1 N HCl. Elemental Pt was measured by FAAS.

The PK parameters were calculated using noncompartmental analysis (WinNonlin standard software, version 3.1; Pharsight Corporation, Palo Alto, CA, USA). The following PK parameters were obtained: AUC, maximum Pt concentration (*C*_max_), time to obtain *C*_max_ (*T*_max_), total body clearance (CL_tot_), terminal half-life of Pt (*t*_1/2_*z*), and steady-state volume of distribution (*V*_ss_). The area under the tumour concentration–time curve (tumour AUC) was calculated based on the trapezoidal rule up to 48 h. The parameters were calculated using the following equations: 
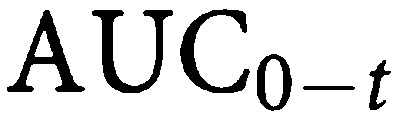
 was calculated by the trapezoidal rule to the last measurable data point: 
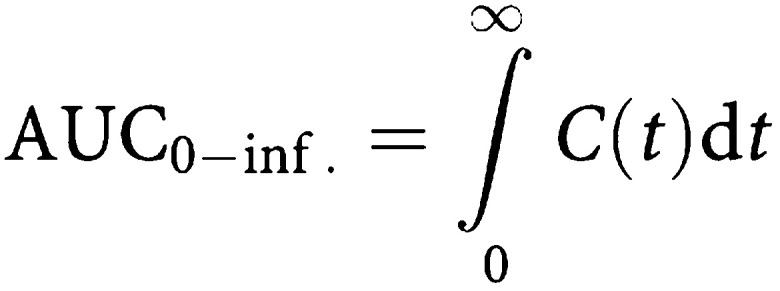


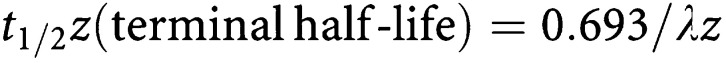

*λ*z: first-order rate constant associated with terminal portion of the curve) 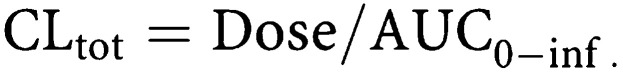


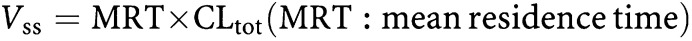


### *In vivo* antitumour activity

Antitumour activity was evaluated using nude mice implanted with a human gastric cancer cell line MKN-4. BALB/*c nu/nu* female mice (aged 6 weeks) were inoculated subcutaneously with 10^6^ MKN-45 cells on the right dorsal skin. After 3 days, when tumour diameter had reached approximately 3 mm, tumour-bearing mice were allocated randomly to drug administration groups of six animals each. The drugs were administered as follows: animals in the CDDP group were given doses of 0.5, 2.5, 5 mg kg^−1^; animals in the NC-6004 group were given doses of 0.5, 2.5, and 5 mg kg^−1^; and animals in the control group were given the 5% glucose solution. Cisplatin or NC-6004 was administered to mice at any of the above dose levels per dose every 3 days. Antitumour activity was evaluated in terms of tumour size by measuring two orthogonal diameters (*a* × *b*: *a*, long diameter; *b*, short diameter) at various time points. Animals were killed by cervical dislocation when the tumour size reached approximately 15 mm (UKCCCR, 1998). Changes in body weight were also monitored for the mice which were used in the present study.

### Nephrotoxicity and hepatotoxicity of CDDP and NC-6004

Under isoflurane anaesthesia, five groups of Sprague–Dawley female rats (aged 6 weeks; 185–215 g initial body weight) were given a single i.v. injection of 5% glucose (*n*=8), CDDP at a dose of 10 mg kg^−1^ (*n*=12), NC-6004 at a dose of 10 mg kg^−1^ on a CDDP basis (*n*=13), or NC-6004 at a dose of 15 mg kg^−1^ on a CDDP basis (*n*=8). Samples of blood and major organs were taken on day 7 after administration (UKCCCR, 1998). In the case of administering NC-6004 at a dose of 10 mg kg^−1^ on a CDDP basis, five samples of blood and major organs were taken on day 14 after administration. The organs were immersed in 10% formalin solution. In each blood sample, plasma concentrations of blood urea nitrogen (BUN), creatinine, glutamic oxaloacetic transaminase (GOT), and glutamic pyruvic transaminase (GPT) were measured by SRL Laboratories (Tokyo, Japan). In addition, WBC and platelet were counted for blood samples 7 and 14 days after each drug administration in SRL Laboratories (Tokyo, Japan).

### Evaluation of neurotoxicity

The severity of neurotoxicity was assessed by electrophysiological and histopathological procedures. Under isoflurane anaesthesia, rats (*n*=5) were given CDDP (2 mg kg^−1^), NC-6004 (an equivalent dose of 2 mg kg^−1^ CDDP), or 5% glucose, all i.v., twice a week, to a total of 11 administrations. Electrophysiological measurements were conducted at week 6 after the first administration, using the method described previously (McKeage *et al*, 1994; Screnci *et al*, 2000). Under light anaesthesia with phenobarbital, responses were evoked by stimulating the sciatic nerve at its notch and the tibial nerve at the ankle of the right hind paw, using a percutaneous needle electrode. The plantar muscle H- and M-waves were recorded using a pair of superficial silver–silver chloride electrodes applied to the sole and dorsum of the hind paw. H-response-related sensory nerve conduction velocity (SNCV) was calculated by dividing the distance between the stimulation sites at the sciatic notch and ankle by the difference in H-response latency after stimulation at the ankle and sciatic notch. M-response-related motor nerve conduction velocity (MNCV) was calculated by dividing the distance between the stimulation sites at the sciatic notch and ankle by the difference in M-response latency after stimulation at the sciatic notch and ankle. At week 7 after the initial administration, rats under deep anaesthesia with phenobarbital were subjected to intracardiac catheterisation and were rinsed with saline, followed by perfusion with 4% glutaraldehyde in 0.12 M PBS. Subsequently, a segment of the sciatic nerve was carefully removed. One part of the sciatic nerve was post-fixed with 4% glutaraldehyde in 0.12 M PBS for 24 h and was then embedded in epoxy resin as described previously (Cavaletti *et al*, 1992). The remaining parts of the sciatic nerve were immersed in a 10% formalin solution. Semi-thin (1 *μ*m thick) and thin sections were prepared from the resin-embedded sciatic nerve for light microscopic observation and electron microscopic observation, respectively.

To determine the Pt concentration in the sciatic nerve, rats were given CDDP (5 mg kg^−1^, *n*=5), NC-6004 (an equivalent dose of 5 mg kg^−1^ CDDP, *n*=5), or 5% glucose (*n*=2), all i.v. twice a week, to a total of four administrations. On day 3 after the final administration, a segment of the sciatic nerve was removed. The removed sciatic nerve was prepared for ICP-MS analysis as described previously (Screnci *et al*, 2000). Briefly, the nerve was immersed in 1 ml of 70% nitric acid overnight. On the next day, the nerve was digested for 2 h at 90°C and Milli-Q was then added to a final volume of 5 ml. Finally, the Pt concentration in the sample solution was analysed with an ICP-MS spectrometer (SPQ 9000; Seiko Instruments Inc., Tokyo, Japan).

### Statistical analysis

Data on therapeutic efficacy and body weight change were expressed as the mean±s.e. The other data were expressed as the mean±s.d. The statistical significance of differences in therapeutic efficacy and body weight change between two administration groups was calculated by repeated-measured analysis of variance (ANOVA). The statistical significance of differences in other data between two administration groups was calculated with the Student’s *t*-test. All data were calculated with StatView® Software, version 5 (ABACUS Concepts, Berkeley, CA). A value of *P*<0.05 was considered statistically significant.

## RESULTS

### Preparation and characterisation of CDDP-incorporating polymeric micelles (NC-6004)

**Figure 1:**
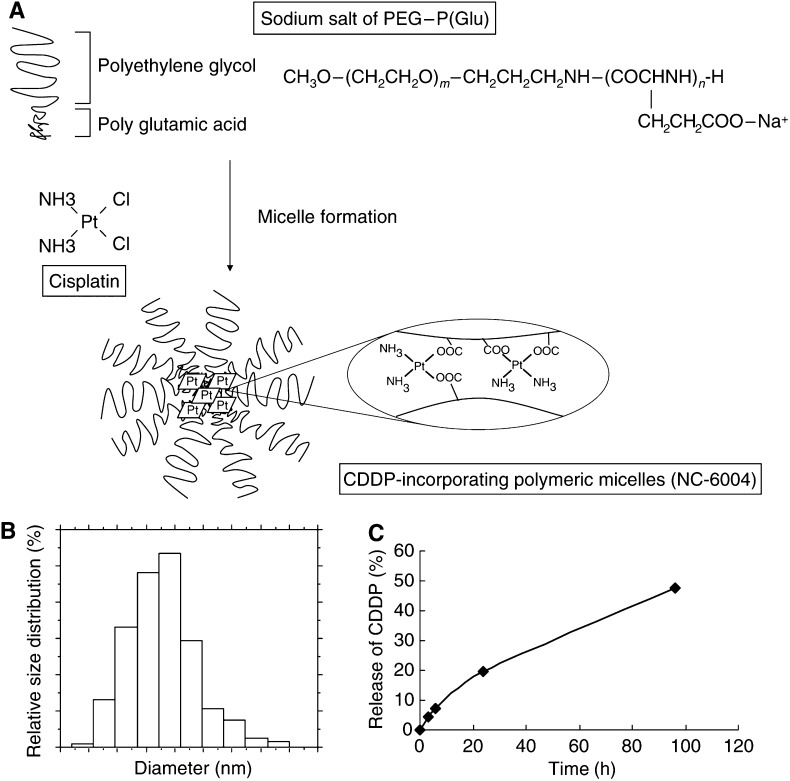
Preparation and characterisation of CDDP-incorporating polymeric micelles (NC-6004). (**A**) Chemical structures of CDDP and PEG–P(Glu) block copolymers, and the micellar structures of CDDP-incorporating polymeric micelles (NC-6004). (**B**) The particle size distribution of NC-6004 measured by the dynamic light-scattering method. The mean particle size of NC-6004 was approximately 30 nm. (**C**) Release of CDDP from NC-6004 in saline at 37°C.

Cisplatin-incorporating polymeric micelles (NC-6004) consist of CDDP and PEG–P(Glu) (Figure 1A). Furthermore, NC-6004 consists of PEG, a hydrophilic chain which constitutes the outer shell of the micelles, and the coordinate complex of P(Glu) and CDDP, a polymer–metal complex-forming chain which constitutes the inner core of the micelles. The molecular weight of PEG–P(Glu) as a sodium salt was approximately 18 000 (PEG: 12 000; P(Glu): 6000). The CDDP-incorporated polymeric micelles were clearly discriminated from typical micelles from amphiphilic block copolymers. The driving force of the formation of the CDDP-incorporated micelles is the ligand substitution of Pt(II) atom from chloride to carboxylate in the side chain of P(Glu). The molar ratio of CDDP to the carboxyl groups in the copolymers was 0.71 (Nishiyama *et al*, 2003). A narrowly distributed size of polymeric micelles (30 nm) was confirmed by the DLS measurement (Figure 1B). Also, the static light scattering (SLS) measurement revealed that the CDDP-loaded micelles showed no dissociation upon dilution and the CMC was less than 5 × 10^−7^, suggesting remarkable stability compared with typical micelles from amphiphilic block copolymers (Nishiyama *et al*. 1999). It is assumed that the interpolymer crosslinking by Pt(II) atom might contribute to stabilisation of the micellar structure.

The release rates of CDDP from NC-6004 were 19.6 and 47.8% at 24 and 96 h, respectively (Figure 1C). Therefore, the release of CDDP was as slow as the previously reported release (Nishiyama *et al*, 2003). In distilled water, furthermore, NC-6004 was stable without releasing CDDP (data not shown).

### Pharmacokinetics and pharmacodynamics

**Figure 2:**
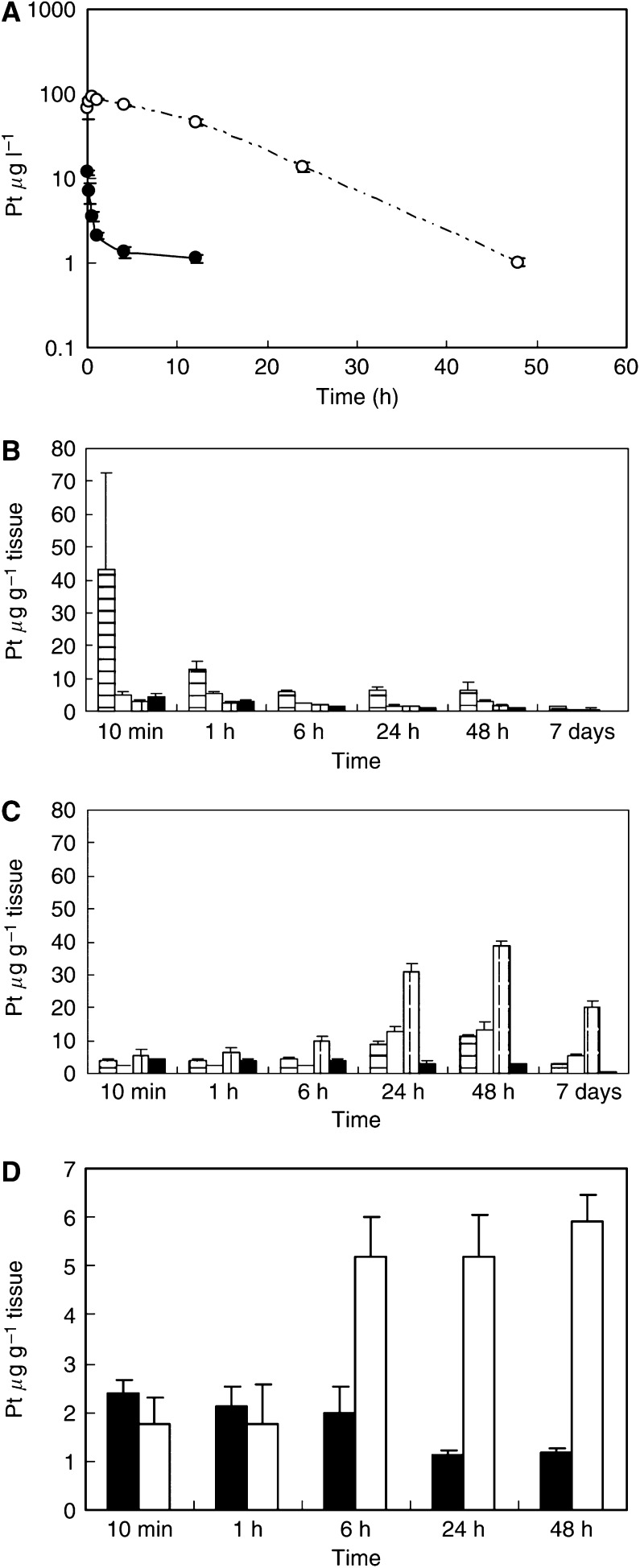
Time profiles of Pt concentration in the plasma and tissue distribution of Pt after a single i.v. injection of CDDP (5 mg kg^−1^) or NC-6004 (an equivalent dose of 5 mg kg^−1^ CDDP). (**A**) Concentration–time profile of Pt in the plasma after a single i.v. injection of CDDP (•) and NC-6004 (○) in rats (*n*=3). Tissue distribution of Pt after a single i.v. injection of CDDP (**B**) and NC-6004 (**C**) in rats (*n*=3) (kidney (
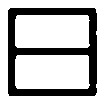
), liver (□), spleen (
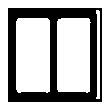
), and lung (▪)). (**D**) Time profiles of Pt concentration in the MKN-45 solid tumour after a single i.v. injection of CDDP (▪) and NC-6004 (□) in MKN-45 bearing BALB/*c* nude mice (*n*=3). Values are expressed as the mean±s.d.

Frameless atomic absorption spectrophotometry could measure serum concentrations of Pt up to 48 h after i.v. injection of NC-6004, but could measure them only up to 4 h after i.v. injection of CDDP. NC-6004 showed a very long blood retention profile as compared with CDDP. The AUC_0−*t*_ and *C*_max_ values were significantly higher in animals given NC-6004 than in animals given CDDP, namely, 65- and 8-fold, respectively (*P*<0.001 and 0.001, respectively) (Table 1, Figure 2A). Furthermore, the CL_tot_ and *V*_ss_ values were significantly lower in animals given NC-6004 than in animals given CDDP, that is, one-nineteenth and one-seventy-fifth, respectively (*P*<0.01 and 0.01, respectively) (Table 1).

Regarding the concentration–time profile of Pt in various tissues after i.v. injection of CDDP or NC-6004, all organs measured exhibited the highest concentrations of Pt within 1 h after administration in all animals given CDDP (Figure 2B). Furthermore, animals given NC-6004 exhibited the highest tissue concentrations of Pt in the liver and spleen at late time points (24 and 48 h after administration, respectively). However, the concentrations decreased on day 7 after administration (Figure 2C). In addition, and in a similar manner to other drugs which are incorporated in polymeric carriers, NC-6004 demonstrated accumulation in organs of the reticuloendothelial system, for example, liver and spleen. At 48 h after administration, tissue concentrations of Pt in the liver and spleen were 4.6- and 24.4-fold higher for NC-6004 than for CDDP. On the other hand, a marked increase in tissue Pt concentration was observed immediately after administration in the kidneys of animals given CDDP. Renal Pt concentration at 10 min and 1 h after administration were 11.6- and 3.1-fold lower, respectively, in animals given NC-6004 than in animals given CDDP. Furthermore, the maximum concentration (*C*_max_) in the kidney was 3.8-fold lower at the time of NC-6004 administration than at the time of CDDP administration.

Regarding the tumour accumulation of Pt, tumour concentrations of Pt peaked at 10 min after administration of CDDP. On the other hand, tumour concentrations of Pt peaked at 48 h after administration of NC-6004 (Figure 2D). The maximum concentration (*C*_max_) in tumour was 2.5-fold higher for NC-6004 than for CDDP (*P*<0.001). Furthermore, the tumour AUC was 3.6-fold higher for NC-6004 than for CDDP (81.2 and 22.6 *μ*g ml h^−1^ in animals given NC-6004 and CDDP, respectively).

### *In vitro* cytotoxicity

NC-6004 was tasted on 12 human tumour cell lines derived from bladder, colon, lung, gastric, and breast cancers. The IC_50_ values of NC-6004 were 6-to 15-fold higher than those of CDDP (Table 2).

### *In vivo* antitumour activity

**Figure 3:**
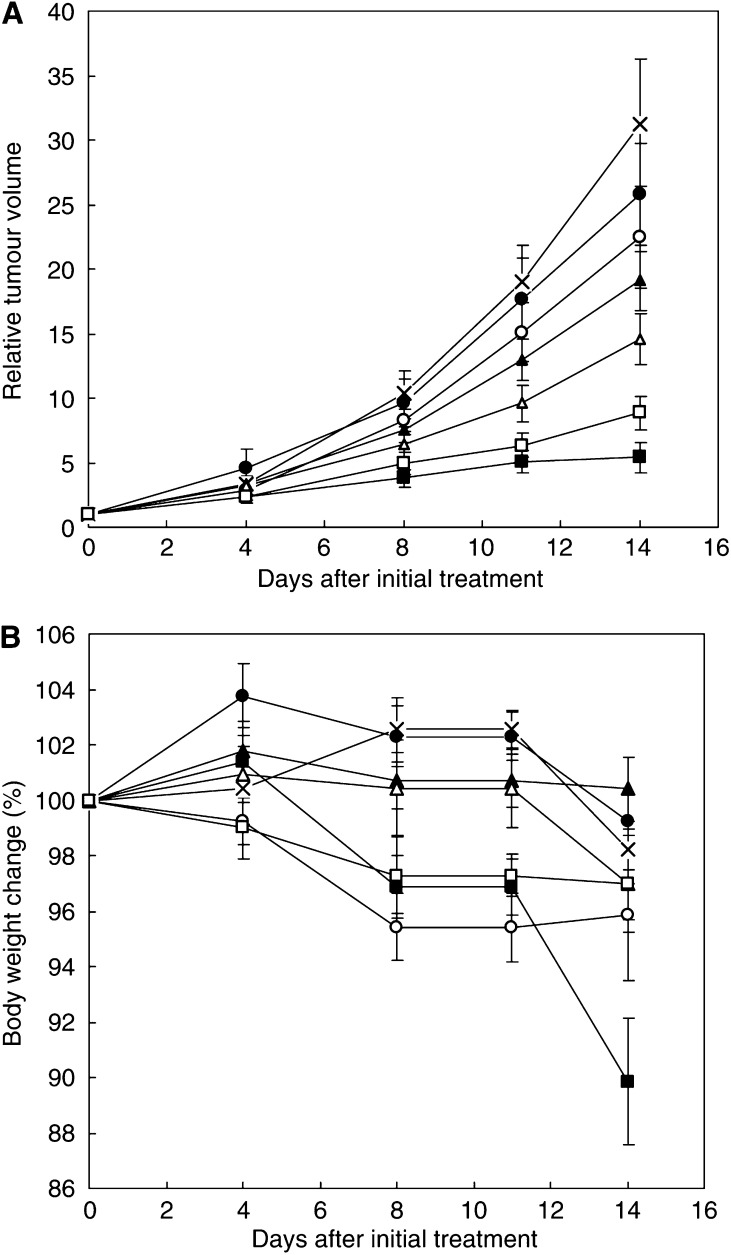
Relative changes in MKN-45 tumour growth rates in nude mice. (**A**) Cisplatin and NC-6004 were injected i.v. every 3 days, three administrations in total, at CDDP-equivalent doses of 0.5 mg kg^−1^ (•, ○), 2.5 mg kg^−1^ (▴, ▵), and 5 mg kg^−1^ (▪, □), respectively. Glucose (5%) was injected in the control mice (×). (**B**) Changes in relative body weight. Data were derived from the same mice as those used in the present study. Values are expressed as the mean±s.e.

BALB/*c* nude mice implanted with a human gastric cancer cell line MKN-45 showed decreased tumour growth rates after i.v. injection of CDDP and NC-6004 (Figure 3A). In the administration of CDDP, the CDDP 5 mg kg^−1^ administration group showed a significant decrease (*P*<0.01) in tumour growth rate as compared with the control group. In the administration of NC-6004, NC-6004 2.5 mg kg^−1^ administration group (*P*<0.05) and 5 mg kg^−1^ administration group (*P*<0.01) showed significant decreases in tumour growth rate as compared with the control group. However, the NC-6004 administration groups at the same dose levels as CDDP showed no significant difference in tumour growth rate. The same animal model was used to repeat the study using the drugs at different dose levels, and similar tendencies were observed (data not shown). Regarding time-course changes in body weight change rate, the CDDP 5 mg kg^−1^ administration group showed a significant decrease (*P*<0.001) in body weight as compared with the control group. On the other hand, none of the NC-6004 administration groups showed a decrease in body weight as compared with the control group (Figure 3B).

### Nephrotoxicity and hepatotoxicity of CDDP and NC-6004

**Figure 4:**
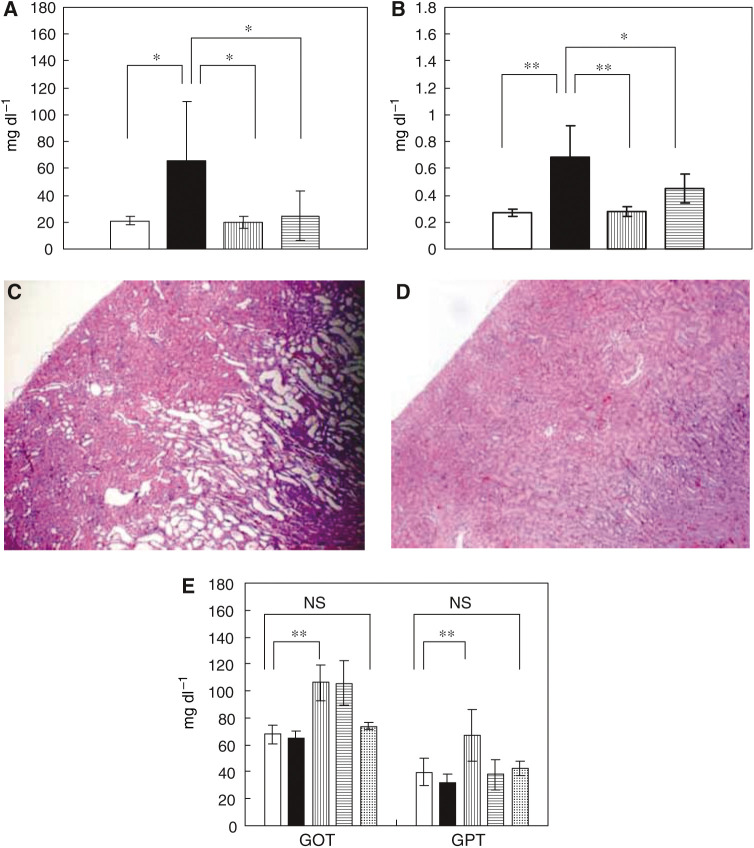
Nephrotoxicity and hepatotoxicity of CDDP and NC-6004. Plasma concentrations of BUN (**A**) and creatinine (**B**) were measured after a single i.v. injection of 5% glucose (□) (*n*=8), CDDP at a dose of 10 mg kg^−1^ (▪) (*n*=12), NC-6004 at a dose of 10 mg kg^−1^ (*n*=13) on a CDDP basis (
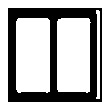
), and at a dose of 15 mg kg^−1^ on a CDDP basis (
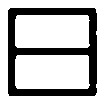
) (*n*=8) to rats. Histopathological changes in the kidney on day 7 after the i.v. injection of CDDP (**C**, × 4) and NC-6004 (**D**, × 4) in rats at an equivalent dose of 10 mg kg^−1^ CDDP. In rats given CDDP, widespread tubular degeneration as indicated by tubular dilation with flattening of the lining cells of tubular epithelium was seen. On the other hand, no histopathological change was observed in the kidney from all animals in the NC-6004 10 mg kg^−1^ administration group. For hepatotoxicity (**E**), the plasma concentrations of GOT and GPT were measured on day 7 after administration. When administering NC-6004 at a dose of 10 mg kg^−1^ on a CDDP basis, five of 13 blood samples were taken on day 14 after administration (
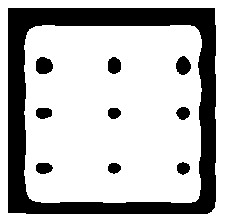
). The other samples were taken on day 7 administration. In the group given CDDP at a dose of 10 mg kg^−1^, four of 12 rats died within 7 days. Values are expressed as the mean±s.d. ^*^P<0.05, ^**^P<0.001, NS: not significant.

In the CDDP 10 mg kg^−1^ administration group, four of 12 animals died from toxicity within 7 days after drug administration. No deaths occurred in the NC-6004 10 mg kg^−1^ administration group and the NC-6004 15 mg kg^−1^ administration group. Regarding renal function, the BUN concentrations on day 7 after the administration of 5% glucose, CDDP 10 mg kg^−1^, NC-6004 10 mg kg^−1^, and NC-6004 15 mg kg^−1^ were 20.8±3.0, 65.3±44.4, 20±4.5, and 24.6±18.2 mg dl^−1^, respectively. The plasma concentrations of creatinine on day 7 after the administration of 5% glucose, CDDP 10 mg kg^−1^, NC-6004 10 mg kg^−1^, and NC-6004 15 mg kg^−1^ were 0.27±0.03, 0.68±0.23, 0.28±0.04, and 0.45±0.11 mg dl^−1^, respectively. The CDDP 10 mg kg^−1^ administration group showed significantly higher plasma concentrations of BUN and creatinine as compared with the control group (*P*<0.05 and 0.001, respectively), with the NC-6004 10 mg kg^−1^ administration group (*P*<0.05 and 0.001, respectively), and also with the NC-6004 15 mg kg^−1^ administration group (*P*<0.05 and 0.05, respectively) (Figure 4A and B). Light microscopy indicated tubular dilation with flattening of the lining cells of the tubular epithelium in the kidney from all animals in the CDDP 10 mg kg^−1^ administration group. On the other hand, no histopathological change was observed in the kidneys from all animals in the NC-6004 10 mg kg^−1^ administration group (Figure 4C and D). Regarding hepatic function, the plasma concentrations of GOT on day 7 after the administration of 5% glucose, CDDP 10 mg kg^−1^, NC-6004 10 mg kg^−1^, and NC-6004 15 mg kg^−1^ were 68±6.8, 65.1±5.5, 106±13.1, and 97±16.2 IU l^−1^, respectively. The plasma concentrations of GPT on day 7 after the administration of 5% glucose, CDDP 10 mg kg^−1^, NC-6004 10 mg kg^−1^, and NC-6004 15 mg kg^−1^ were 39.6±10, 32±6.4, 92±18.9, and 55±11.3 IU l^−1^, respectively. The CDDP 10 mg kg^−1^ administration group showed plasma concentrations of GOT and GPT which were comparable to those in the control group. However, the NC-6004 10 mg kg^−1^ administration group, which presented the same dose level as the CDDP 10 mg kg^−1^ administration group, showed significantly higher plasma concentrations of GOT and GPT (*P*<0.001 and 0.01, respectively) as compared with the control group. Furthermore, the NC-6004 15 mg kg^−1^ administration group also showed significantly higher plasma concentrations of GOT (*P*<0.001) as compared with the control group. However, the plasma concentrations of GOT and GPT on day 14 after the administration of NC-6004 10 mg kg^−1^ were comparable to those in the control group (74±2.3 and 42.8±5.1 IU l^−1^, respectively) (Figure 4E). These results lead to the conjecture that rats which were given NC-6004 10 mg kg^−1^, i.v., showed transient and reversible hepatotoxicity.

### Neurotoxicity of CDDP and NC-6004

**Figure 5:**
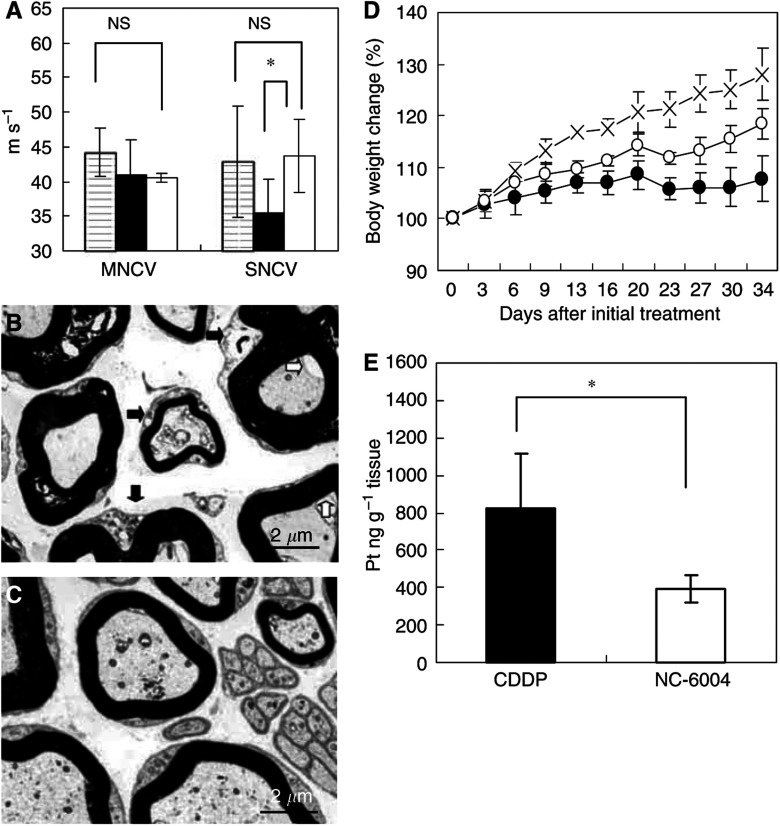
Neurotoxicity of CDDP and NC-6004 in rats. Rats (*n*=5) were given CDDP (2 mg kg^−1^), NC-6004 (an equivalent dose of 2 mg kg^−1^ CDDP), or 5% glucose, all i.v. twice a week, 11 administrations in total. (**A**) Sensory nerve conduction velocity and MNCV of the sciatic nerve at week 6 after the initial administration (control (
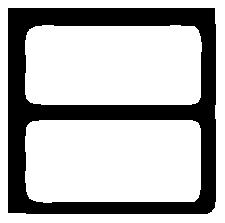
), CDDP (•), and NC-6004 (□)). Histopathological changes of the sciatic nerve were examined by electron microscopy after the administration of CDDP (**B**) and NC-6004 (**C**). In rats given CDDP, widespread degenerations as indicated by loss of microtubules, loss of filaments, degeneration in the cytoplasm of Schwann cells (
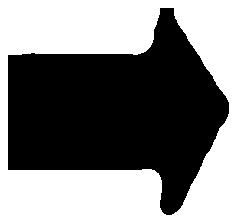
), and an irregular inner loop (
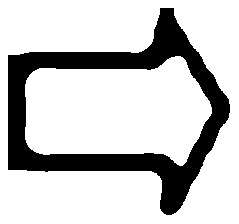
) were seen. On the other hand, animals given NC-6004 exhibited nearly normal electron micrographs of the sciatic nerve as the control animals. (**D**) Changes in relative body weight. Data were derived from the same rats as those used in the present study (control (×), CDDP (•), and NC-6004 (○)). (**E**) The Pt concentration in the sciatic nerve. Rats were given CDDP (▪) (5 mg kg^−1^, *n*=5), NC-6004 (□) (an equivalent dose of 5 mg kg^−1^ CDDP, *n*=5), or 5% glucose (*n*=2), all i.v. twice a week, four administrations in total. On day 3 after the final administration, a segment of the sciatic nerve was removed and the Pt concentration in the sciatic nerve was measured by ICP-MS. Body weight changes are expressed as the mean±s.e. The other data are expressed as the mean±s.d. ^*^P<0.05, ^**^P<0.001, NS: not significant.

Neurophysiological examination revealed that MNCVs in animals given 5% glucose, CDDP, and NC-6004 were 44.2±3.5, 40.94±5.08, and 40.62±0.63 m s^−1^, respectively. No significant difference was found among the groups with respect to MNCV. Furthermore, SNCVs in animals given 5% glucose, CDDP, and NC-6004 were 42.86±8.07, 35.48±4.91, and 43.74±5.3 m s^−1^, respectively. Animals given NC-6004 showed no delay in SNCV as compared with animals given 5% glucose. On the other hand, animals given CDDP showed a significant delay (*P*<0.05) in SNCV as compared with animals given NC-6004 (Figure 5A). In addition, histopathological examination with electron microscopy revealed degenerations, as manifested by electron photomicrographs indicating degenerative changes, for example, loss of microtubules, degeneration in the cytoplasm of Schwann cells, loss of filaments, and an irregular inner loop, in approximately 80% of myelinated segments of the sciatic nerve from animals given CDDP. On the other hand, animals given NC-6004 exhibited nearly normal electron photomicrographs of the sciatic nerve as the control animals did (Figure 5B and C). These results indicate that NC-6004 reduced peripheral neurotoxicity as compared with CDDP. Furthermore, regarding body weight change as an indication of general toxicity, furthermore, the NC-6004 administration groups showed significant inhibition of body weight decrease (*P*<0.001) as compared with the CDDP administration group (*P*<0.001) (Figure 5D).

The analysis by ICP-MS on sciatic nerve concentrations of Pt could not detect Pt in the sciatic nerve from animals given 5% glucose (data not shown). Sciatic nerve concentrations of Pt in animals given CDDP and NC-6004 were 827.2±291.3 and 395.5±73.1 ng g^−1^ tissue. Therefore, the concentrations were significantly (*P*<0.05) lower in animals given NC-6004 (Figure 5E). This finding is believed to be a factor which reduced neurotoxicity following NC-6004 administration as compared with the CDDP administration.

## DISCUSSION

The present study indicated that CDDP-incorporating polymeric micelles (NC-6004) are stable nanoparticles with a long blood retention profile as compared with free CDDP. NC-6004 showed 6- to 15-fold less potent *in vitro* cytotoxic activity in several human cancer cell lines as compared with CDDP. These findings are considered attributable to the slow release of free CDDP in the presence of abundant chloride ions because NC-6004 contains coordination bonds between the atoms of Pt(II) of CDDP and the carboxylic group in the side chain of P(Glu). *In vivo*, however, in contrast to the *in vitro* findings, NC-6004 was found to markedly reduce nephrotoxicity and neurotoxicity – dose-limiting factors of CDDP, while preserving antitumour activity, which was equivalent to or better than that of free CDDP.

Nephrotoxicity of CDDP is considered to depend on the peak urinary CDDP concentration and on the maximum CDDP concentration in the uriniferous tubules (Levi *et al*, 1982). We consider that the reduced nephrotoxicity of NC-6004 may be explained by the following facts: (1) the tendency of micelles to be less prone to filtration by nephrons because of the NC-6004 particle size (approximately 30 nm), and (2) the much lower *C*_max_ value for CDDP at least in the uriniferous tubules than the value following CDDP administration. NC-6004 possibly facilitates treatment on an outpatient basis because it allows safer administration to patients with decreased renal function and requires no massive fluid replacement to protect renal tissue after the administration of CDDP.

The main neuropathy of CDDP is sensory peripheral neuropathy (van der Hoop *et al*, 1990; Gregg *et al*, 1992). A delay in SNCV due to the injury of dorsal root ganglia and peripheral nerve has previously been reported in rats given CDDP, although MNCV was preserved in the tail and hind paws of rats (McKeage *et al*, 1994; Tredici *et al*, 1998; Meijer *et al*, 1999; Tredici *et al*, 1999). Furthermore, histopathological examination revealed degenerative changes in the sciatic nerve in similar experimental animals (Cavaletti *et al*, 1992; Tredici *et al*, 1999). In the present study, animals given NC-6004 showed no delay in the SNCV, while animals given CDDP showed a significant delay in the SNCV as compared with animals given NC-6004. Neuropathologically, neuronal degeneration, which was observed following CDDP administration, was not observed with NC-6004 administration. This result is considered attributable principally to the fact that the peripheral nerve concentration of Pt decreased to half or less following NC-6004 administration than with CDDP administration. The nervous tissue concentration of Pt at the time of NC-6004 administration decreased significantly despite the fact that the plasma AUC at the time of NC-6004 administration was high, being 65-fold higher than the plasma AUC concentration with CDDP administration. We consider that this result is attributed to the marked inhibition of Pt distribution into nervous tissue in the NC-6004 administration groups as manifested by *V*_ss_ of 3.00±0.61 and 0.04±0.0023 l kg^−1^ in the CDDP and NC-6004 groups, respectively. In any event, we believe that the neurotoxicity of CDDP reduced by NC-6004 allows its long-term administration.

On the other hand, transient hepatic dysfunction was observed in rats. This observation indicates the proneness of Pt to accumulate in the RES of the liver because NC-6004 is, after all, said and done, a macromolecule, although preserving a stealth effect through its outer shell of PEG. We consider that caution should be exercised against hepatic dysfunction in conducting a clinical trial of NC-6004 in the future. However, the accumulation of Pt was lower following NC-6004 administration due to a decrease in *V*_ss_ in other organs including nerve. As shown by changes in body weight in multiple dose studies in rats, the NC-6004 administration groups have been demonstrated to show a smaller decrease in body weight as compared with the CDDP administration groups. In single-dose studies, furthermore, one dose of CDDP 10 mg kg^−1^ was equivalent to the 50% of the lethal dose. In fact, four of 12 animals died within 7 days after administration. However, none of the eight animals in the NC-6004 group died after the administration of NC-6004 at a CDDP equivalent dose of 15 mg kg^−1^. In terms of haematological toxicity, there was no significant difference between the CDDP and NC-6004 groups in rats (data not shown).

In murine tumour strains, CDDP-incorporating polymeric micelles showed significantly high antitumour activity (Nishiyama *et al*, 2003). In the human gastric cancer strain used in the present study, however, no significant difference was found between the NC-6004 and CDDP administration groups. A significant difference was found in antitumour activity between the NC-6004 low-dose group (2.5 mg kg^−1^ administration group) and the control group, while no significant difference was found between the CDDP low-dose group (2.5 mg kg^−1^ administration group) and the control group. Results available to date and the results from the present study lead to the consideration that the incorporation of CDDP into polymeric micelles does not reduce its antitumour activity.

Data from the present study warrant the clinical evaluation of NC-6004. We consider that the protocol for the Phase I clinical trial of NC-6004 should employ a regimen without massive i.v. drip infusion.

## Figures and Tables

**Table 1:** Pharmacokinetic parameter estimates for CDDP and NC-6004 in rats (see text for definitions of parameters)

**Compound**	**Rat**	***T*_max_^a^ (h)**	***C*_max_^a^ (*μ*g ml^−1^)**	***t*_1/2_*z* (h)**	**AUC_0–*t*_ (*μ*g h ml^−1^)**	**AUC_0–inf._ (*μ*g h ml^−1^)**	**CL_tot_ (ml h^−1^ kg^−1^)**	**MRT_0–inf._ (h)**	***V*_ss_ (l kg^−1^)**
CDDP	Mean s.d.	0.083	11.67	34.50	20.47	75.73	70.67	46.57	3.00
			0.57	16.14	2.25	26.13	20.34	22.38	0.61
									
NC-6004	Mean s.d.	0.50	89.90	6.43	1325.90	1335.47	3.77	10.67	0.04
			4.29	0.55	77.85	75.99	0.21	0.15	0.0023

The pharmacokinetic parameters were calculated after fitting to a noncompartment model using WinNonlin program.

a For CDDP group, *T*_max_ represents time of maximum concentration.

**Table 2:** IC_50_ values(*μ*M) of CDDP and NC-6004 in various cancer cell lines

		**Exposure time (h)**			
		**48**		**72**	
**Cancer**	**Cell line**	**CDDP**	**NC-6004**	**CDDP**	**NC-6004**
Bladder cancer	EJ-1	2.46	25.45	1.86	18.44
	J82	2.78	42.89	2.42	20.27
	MBT-2	15.88	>100	5.67	71.67
Colon cancer	Colo201	34.77	>100	28.52	>100
	Colo320	16.32	>100	9.71	81.15
	HT-29	14.44	>100	8.83	>100
Lung cancer	A549	21.43	>100	20	>100
	EBC-1	>100	>100	9.36	84.78
	PC-14	16.81	>100	8.73	87.11
Gastric cancer	MKN-28	>100	>100	8.23	76.81
	MKN-45	7.12	68.36	6.94	43.81
Breast cancer	MCF-7	12.78	>100	5.71	54.71
